# Trends in child marriage, sexual violence, early sexual intercourse and the challenges for policy interventions to meet the sustainable development goals

**DOI:** 10.1186/s12939-023-02060-9

**Published:** 2023-12-05

**Authors:** Kathya Lorena Cordova-Pozo, Sujata Santosh Anishettar, Manish Kumar, Praveen Kailash Chokhandre

**Affiliations:** 1 Institute for Management Research, Radboud University, Nijmegen, The Netherlands; 2 Family Planning Association of India, Dharwad, India; 3 Population Research Centre, JSS Institute of Economic Research Center, Dharwad, Karnataka India

**Keywords:** Child marriage, Sexual violence, Early sexual intercourse, Adolescents, Low- and middle-income countries, Policy interventions, Sustainable development goals

## Abstract

**Introduction:**

Child marriage remains a prevalent issue in low- and middle-income countries (LMIC) despite global declines. Girls are disproportionately affected, facing health risks, limited education, and restricted decision-making power. We aim to provide insights for child marriage prevalence across LMIC from 1990 to 2020, with a focus on sexual violence and early sexual intercourse for public health policy interventions.

**Methods:**

This study used World Bank datasets to assess progress in addressing child marriage in LMIC countries. Statistical analyses, including trend analysis and compound annual growth rate (CAGR), were conducted to evaluate indicators of first marriage, sexual violence, and sexual intercourse. Countries with sufficient data were categorized based on prevalence rates and trends, and detailed analysis focused on significant indicators.

**Results:**

While significant reductions were observed in the prevalence of child marriage before the age of 15 and 18 and early sexual intercourse in most countries, few countries show increasing trends, and others could not demonstrate statistical trends due to data limitations, such as scarcity of data for boys. Overall, many countries showed a decline in sexual violence and early sexual intercourse before the age of 15, but some exhibited increasing trends. For instance, Zambia and Senegal showed a decreasing trend of sexual violence, while Nigeria exhibited an increasing trend. Notably, Uganda, Cameroon, and Sierra Leone for women, and Namibia, Zambia, and Kenya for men, experienced substantial decline in early sexual intercourse.

**Conclusion:**

There is a decline in child marriage, sexual violence, and early sexual intercourse in most countries independent from the income group. Only a few countries show slight increasing trends. The improvements confirm that policies that address education, employment, and deep-rooted gender inequality at the societal level seem to be effective and help reach the SDG. However, better data are needed to enhance the understanding of the development of child marriage in these countries to improve the effectiveness of policy intervention. Therefore, we recommend that policymakers not only include existing evidence that continues progress but also increase and improve the monitoring of relevant indicators.

**Supplementary Information:**

The online version contains supplementary material available at 10.1186/s12939-023-02060-9.

## Plain Language Summary

Child marriage remains a prevalent issue despite global declines, particularly affecting girls who suffer from health risks, lower education, and restricted decision-making power. However, little is known about boys in this context. This study aims to examine We aim to provide insights for child marriage prevalence across LMIC from 1990 to 2020, with a focus on sexual violence and early sexual intercourse for public health policy interventions. To achieve this, we assessed progress in addressing child marriage in LMIC countries using World Bank datasets. Through statistical analyses, including trend analysis and linear regression, we evaluated various indicators. Countries with sufficient data were categorized based on prevalence and trends. The results revealed significant reductions in all indicators, few countries show increasing trends, and others could not demonstrate statistical trends due to data limitations, such as scarcity of data for boys. The Central African Republic experienced an increase in child marriage prevalence. Overall, many countries showed a decline in sexual violence and intercourse before the age of 15, but some exhibited increasing trends. For instance, Zambia and Senegal showed a decreasing trend of sexual violence, while Nigeria exhibits an increasing trend. Notably, Uganda, Cameroon, and Sierra Leone experienced substantial declines in early sexual intercourse for women, and Namibia, Zambia, and Kenya for men. There is a decline in child marriage, sexual violence, and early sexual intercourse in most countries independent of the income group. Only a few countries show slight increasing trends. The improvements confirm that policies that address education, employment, and deep-rooted gender inequality at societal level seem to be effective and help reach the SDG. However, better data are needed to enhance the understanding of the development of child marriage in these countries to improve the effectiveness of policy intervention. Therefore, we recommend that policymakers not only include existing evidence that continues progress but also increase and improve the monitoring of relevant indicators.

**Supplementary Information:**

The online version contains supplementary material available at 10.1186/s12939-023-02060-9.

## Introduction

Child marriage refers to formal or informal union before the age of 18 [49]. This practice constitutes a violation of human rights, depriving individuals of a life free from all forms of violence and having severe consequences on well-being and rights [52]. Alarmingly, each year, nearly 12 million girls worldwide are married as children [52]. If efforts to prevent child marriage are not accelerated, it is estimated that over 120 million more girls will be married as children by 2030, according to UNICEF. Despite global efforts, the decline in the child marriage rates is uneven across regions, countries, and even within countries [50]. The UNFPA-UNICEF Global Program to End Child Marriage was launched in 2016, specially targeting high-prevalence countries [51] by using a multi-sectoral approach engaging with education, child protection, and social protection sectors, and using social and behavioral change, this program has shown a positive outcome for the girls. Child marriage disproportionately affects girls, with one in seven being married before the age of 15, and one in three before the age of 18 [11, 55]. A study by Wodon et al. [54] has revealed the severe consequences of child marriage for girls, including increased health risks, fertility problems, and intimate partner violence. Moreover, it may lead to lower educational attainment, reduced earnings, and diminished decision-making power within the household.

Girls from rural and disadvantaged backgrounds in low- and middle-income countries (LMIC) are particularly vulnerable to the negative effects of child marriage, perpetuating cycles of poverty and inequality [3, 31, 44]. Often forced into marriage, these girls have little control over when or whom they marry, leaving them at higher risk of experiencing domestic violence [22, 36]. Furthermore, they may lack access to safe sex and medical care, making them susceptible to health risks like sexually transmitted infections and child pregnancies [8, 20]. Which can lead to adverse pregnancy outcomes for both the young mothers [2, 17, 18, 42] and their children [11]. While data on child marriage among boys is limited, it is crucial to observe these trends and their consequences. Boys who marry at a young age may also face significant limitations in educational and career opportunities, perpetuating poverty, and resulting in negative social and psychological outcomes, such as stress, anxiety, and social isolation, impacting their well-being and ability to engage in their communities [5, 54].

Over the years, the incidence of child marriage has decreased in all income groups countries, with a global decline in prevalence from 25 in 1990 to 19 in 2017 [29]. This decrease is particularly notable in South Asia, with 24 between 2000 to 2017 [29]. However, despite the strategies and interventions, child marriage remains a prevalent issue and there are significant disparities between countries. In Sustainable Development Goal (SDG) 5, Target 5.3 aims to eliminate all harmful practices, including child, early and forced marriage, by 2030 [27]. Numerous studies indicate that social norms and attitudes play a crucial role in determining child marriages. Usually, parents make the decision, influenced by their attitudes and community norms [1, 13, 43].

Although individual agency to delay marriage is important, some communities lack access to information about the negative consequences of child marriage [23, 33]. In certain communities, the value and protection of girls' virginity are deeply rooted in religious norms and expectations of purity [19]. Additionally, in some conservative settings, an earlier age at menarche is associated with a younger age at marriage, potentially as a strategy to avoid pregnancy outside of marriage [16, 26]. Moreover, in some distressing cases, early pregnancy may force marriage, even when the pregnancy is a result of rape [53]. The analysis in this study focuses on the patterns observed among adolescents who experienced child marriage by 15 or 18 years, sexual violence before the ages of 15, 18, and 22, and early sexual intercourse before 15 years. The intention is not to attach a negative connotation to the age of sexual intercourse but rather to provide an indication of the prevailing situation in line with the statements.

We aim to describe the prevalence changes in child marriage across and the other indicators within LMIC over a 30-year period (1990–2020) to provide insights for programs that employ a multisectoral approach and focus on behavioral change, along with contributing to the development of both national and local public health policy interventions.

## Methods

### Data

We included several indicators, the percentage of women aged 20–24 who were first married by age 15 and by age 18, the percentage of women age 15–49 who experienced first sexual violence before age 15, 18, and 22; and the percentage of adolescents aged 15–19 who initiated sexual intercourse before age 15. The prevalence of these incidators in all LMIC was assessed using data from the World Bank Statistics datasets. It is important to note that the data for men were only available for the last indicator.

### Statistical analysis

To assess the trends of the indicators mentioned, the methodologogy involved was time trend analysis of the prevalence with the hypothesis of trend and no trend under a p-value threshold set at P-value ≤ 0.10. The analysis included only countries with three or more data points between 1990 and 2023 to estimate trends in child marriage. We employed trend analysis through linear regression to evaluate the projected child marriage trends in different countries. The formula for simple linear regression is as follows:

1 $$\mathrm{Y }=\upbeta 0 +\upbeta 1*\mathrm{X }+\mathrm{ e}$$

Where Y is the dependent variable, X is the independent variable, b0 is the intercept, b1 is the slope or the coefficient of X, and e is the error term or the difference between the actual value of Y. Due to a lack of data for several years, p-values up to 0.10 were included. After trendlines were calculated and drawn, the change of percentage was calculated. The formula used for this calculation was:

2 $$C= \frac{{y}_{0}-{y}_{\mathrm{e}}}{{y}_{\mathrm{e}}}$$

Where C is the relative change, ${\mathrm{y}}_{0}$ is the initial value, and ${\mathrm{y}}_{e}$ is the final value.

Compound annual growth rates (CAGRs) were computed to assess average yearly changes of prevalence [45]. It is usually used in finance to quantify the growth as a metric over time and serves as a comparator to the trend analysis adding the magnitude of growth per year.

### Categorizing countries based on data and prevalence criteria

We carefully observed the countries that either lacked any data or had less than 2 data points, especially focusing on those with the highest prevalence rates in the past or present, as no clear trend could be discerned (Additional file 1: Appendix 5). Additionally, countries with a non significant value (p-value > 0.10) were included to highlight those that might be left behind due to inadequate monitoring of the situation over time. To categorize these countries, we used the prevalence rate (y_e_), selecting the upper 50 as the top countries deserving more attention and assistance in efforts to reduce child marriage rates.

A detailed analysis was performed in those with a significant p-value, e.g. the initial and current prevalence value (y_0,_ y_e_) respectively, average year change (μ), and coefficient values (β) within the trend. To ensure meaningful analysis and comparability, we classified the indicators into distinct categories of low, medium, and high, based on their highest values, and then divided them into three equal groups (Table1). The top countries with the highest coefficient values (β) and the highest prevalence rates (y_e_) were presented.

**Table 1:** Categorization of the indicators used

**Indicators**	**Category based on Prevalence rate**		
	**LOW**	**MEDIUM**	**HIGH**
Women who were first married by age 15 (% of women ages 20–24)	0–9.3	9.4–18.7	18.8–28.0
Women who were first married by age 18 (% of women ages 20–24)	0–25.4	25.5–50.9	51–76.3
Women who experienced first secual violence before age 15 (% of women ages 15–49)	0–2.1	2.2–4.3	4.4–6.4
Women who experienced first secual violence before age 18 (% of women ages 15–49)	0–3.9	4–7.8	7.9–11.7
Women who experienced first secual violence before age 22 (% of women ages 15–49)	0–6.1	6.2–12.1	12.2–18.2
Women who initiated sexual intercourse before age 15 (% of women ages 15–19)	0–8.2	8.3–16.3	16.4–24.5
Men who initiated sexual intercourse before age 15 (% of men ages 15–19)	0–11.7	11.7–23.2	23.3–34.8

## Results

This study aimed to assess progress in addressing child marriage, sexual violence, and age at first intercourse in 137 LMIC countries. For this, we present the trends of the past 30 years.

### Child marriage at 15 or at 18 years

The analysis indicates progress in reducing child marriage before the age of 15 years over the last three decades; however, this progress is uneven (Table 3). Among the 137 LMIC countries, 32 countries have less than 2 data points for this indicator. Out of the 105 countries with two or more data points, 17 countries show a non-significant trends. Conversly, 88 countries display a clear trend, with 26 countries demonstrating an increasing trend, while 62 countries showcase a decreasing trend. (Additional file 1: Appendix 5).

#### Highest prevalence

Three decades ago, Niger (y_1992_; 50), Bangladesh (y_1994_; 47), and Mali (y_1987_; 27) had the highest rates of child marriage. These child marriage rates have, on average, reduced by 43 within the high and medium prevalence groups and by 29 in the low prevalence category. Currently, Niger, Central African Republic, Chad, Mauritania, and Guinea have a higher prevalence of child marriage (Table 2). Among the countries with the highest and medium prevalence, significant progress has been made, as observed in Cameroon (y_0_ = 21, y_e_ = 11), and Yemen (y_0_ = 20, y_e_ = 9), where child marriage rates have considerably decreased over time, transitioning from high to low prevalence. Additionally, some countries with medium prevalence have successfully reduced their rates to low levels, including Sierra Leone, Nepal, Uganda, India, Liberia, Nicaragua, Senegal, Malawi, Cote d'Ivoire, and Guatemala, while others like the Central African Republic has increased over time (Additional file 1: Appendix 1).

**Table 2:** Highest prevalence rates for women who were first married by age 15 or 18

Country	Income Category	Prevalence rate in	Year	Category based on the Prevalence
**- Marriage by 15**				
Niger	Low	28,0	2012	high
Central African Republic	Low	25,8	2019	high
Chad	Low	24,2	2019	high
Mauritania	Low-middle	17,8	2015	medium
Guinea	Low	17,0	2018	medium
**- Marriage by 18**				
Niger	Low	76,3	2012	high
Central African Republic	Low	61,0	2019	high
Chad	Low	60,6	2019	high
Mali	Low	53,7	2018	high
Mozambique	Low	52,9	2015	high

#### Trends

Among the countries that are experiencing an increasing trend in child marriage rates, the highest coeficient values (β) are observed in Somalia, Belize, and Papua New Guinea, with medium and low prevalence values (y_e_). Conversly, countries with the largest decreasing coefficients are Afghanistan, Bangladesh, and Niger with low, medium and high prevalence respectively. Specifically, Central African Republic, Madagascar, and Somalia have the highest rates in the increasing trend, while Niger, Mauritania, and Guinea are among the countries with decreasing trend. Seventeen countries show a non-significant value, indicating no clear trend over time. Chad, Burkina Faso, and Congo have the highest prevalence rates, but show no trends in the past 30-years (Table 3).

**Table 3:** Highest coefficients and rates of women who were first married by 15 (of women ages 20–24), p-value ≤ 0.10

Country	Income Category	Coefficient	p-val	Sig	Start Rate (yo)	End Rate (ye)	Start Year	End Year	Category based on end value
**Increasing trend** ^a^									
Somalia	Low	**0.6**	0	*	8.4	16.8	2006	2020	medium
Belize	Upper-middle	**0.58**	0	*	3.4	6.3	2011	2016	medium
Papua New Guinea	Low-middle	**0.49**	0	*	2.1	8.0	2006	2018	low
Central African Republic	Low	0.3	0.09	*	**19.6**	**25.8**	1995	2019	high
Madagascar	Low	0.14	0.06	*	8.6	12.7	1992	2018	medium
**Decreasing trend** ^a^									
Afghanistan	Low	**-2.3**	0	*	8.8	4.2	2015	2017	low
Bangladesh	Low-middle	**-1.25**	0	*	47.2	15.5	1994	2019	medium
Niger	Low	**-0.95**	0.01	*	50.3	28.0	1992	2012	high
Mauritania	Low-middle	-0.21	0	*	20.8	17.8	2001	2015	medium
Guinea	Low	-0.42	0.01	*	27.5	17.0	1999	2018	medium
**No trend** ^b^									
Chad	Low	-0.22	0.19		28.6	24.2	1997	2019	high
Burkina Faso	Low	0.01	0.94		9.5	10.2	1993	2010	medium
Congo, Dem. Rep	Low	0.03	0.72		8.3	8.4	2007	2018	low

^a^The top three countries with the highest coefficient rates, and the top three countries with the highest prevalence rates. There is an overlap between the highest coefficient and highest prevalence rates, and thus, there are only five countries listed

^b^Only the top prevalence rates were included as the coefficients were non-significant to statistically show a trend

In terms of marriage before the age of 18 years, many countries have shown significant progress towards reducing it (Table 4). Out of the 137 LMIC countries, 27 countries have less than 2 data points for this indicator. Among the 110 countries with available data, 17 countries have a non-significant p-value, suggesting no discernible trend, while 93 show a trend: 17 of which have an increasing trend and 76 a decreasing one (Additional file 1: Appendix 5).

**Table 4:** Highest coefficients and rates of women who were first married by 18 (of women ages 20–24), p-value ≤ 0.10

Country	Income Category	Coefficient	p-val	Sig	Start Rate (yo)	End Rate (ye)	Start Year	End Year	Category based on end value
**Increasing trend** ^a^									
Suriname	Upper-middle	**1.52**	0.01	*	19.1	36.0	2006	2018	medium
Belize	Upper-middle	**1.52**	0	*	25.9	33.5	2011	2016	medium
Iraq	Upper-middle	**0.82**	0.02	*	17.0	27.9	2006	2018	medium
Dominican Republic	Upper-middle	0.2	0.02	*	34.4	35.9	1986	2014	medium
Sudan	Low	0.31	0	*	26.9	34.2	1990	2014	medium
**Decreasing trend** ^a^									
Sierra Leone	Low	**-1.89**	0	*	55.6	29.6	2005	2019	medium
Afghanistan	Low	**-1.37**	0	*	39.0	28.3	2008	2017	medium
Nepal	Low-middle	**-1.23**	0	*	60.3	32.8	1996	2019	medium
Niger	Low	-0.35	0.03	*	83.5	76.3	1992	2012	high
Chad	Low	-0.44	0	*	71.4	60.6	1997	2019	high
Mali	Low	-0.81	0	*	78.1	53.7	1987	2018	high
**No trend** ^b^									
Central African Republic	Low	0.28	0.33		57.0	61.0	1995	2019	high
Madagascar	Low	0.17	0.16		36.9	40.3	1992	2018	medium
Mauritania	Low-middle	-0.06	0.56		37.2	37.0	2001	2015	medium

^a^The top three countries with the highest coefficient rates, and the top three countries with the highest prevalence rates

^b^Only the top prevalence rates were included as the coefficients were non-significant to statistically show a trend

#### Highest prevalence

Three decades ago, the highest rates were observed in Niger (y_1992_ = 83), Mali (y_1987_ = 78), and Bangladesh (y_1994_ = 73). On average, these rates have decreased by 13 within the high and medium prevalence groups and by 21 in the low prevalence category. This translates to a reduction from 16 to 6 countries with high prevalence category and from 37 to 34 countries with medium parevalence category. The highest rates are found in Niger, Central African Republic, Chad, Mali, and Mozambique (Table 2). Some countries have transitioned from high to medium prevalence, such as Uganda (Low income, y_0_ = 53, y_e_ = 34), Cameroon (y_0_ = 58, y_e_ = 30), and Sierra Leone (y_0_ = 56, y_e_ = 30). Conversly, there are countries that have shifted from low to medium prevalence, including Suriname (y_0_ = 19, y_e_ = 36), Sudan (y_0_ = 27, y_e_ = 34), Iraq (y_0_ = 17, y_e_ = 28), and Papua New Guinea (Low-middle income, y_0_ = 21, y_e_ = 27) (Additional file 1: Appendix 2).

#### Trends

Countries with an increasing trend and the highest coeficient values (β) for child marriage rates are Suriname, Belize, and Iraq, with medium prevalence values (y_e_). Conversly, countries with the largest reduction coefficients are Sierra Leone, Afghanistan, and Nepal. Sierra Leone, Cameroon, and Eritrea have transitioned from high prevalence to a medium–low rate over the past 27 years.

Among the countries with the highest rates and an increasing trend in child marriage are the Dominican Republic, and Sudan, while countries with a decreasing trend include Niger, Chad, and Mali. Seventeen countries show a non-significant value, indicating no clear trend over time. The highest prevalence rates are found in the Central African Republic, Madagascar and Mauritania, while the other rates, ≤ 33.66, are categorized as medium (Table 4).

In summary, the results reveal distinct patterns for marriage before the age of 15 and 18 years. First, the average prevalence for marriage by age 15 reduced from 23% in ≈1994 to 19% ≈2016, and the marriage by age 18 reduced from 59% in to 43% among the top 20 countries. Niger, Central African Republic and Chad have the highest prevalence rates for child marriage by age 15 and 18. Second, marriage by age of 15 have a current prevalence that labels 3 countries with high, 18 with medium and 84 with low prevalence while marriage by age 18 labels 7 countries with high, 39 with medium and 64 with low prevalence. Additionally, 23 and 24 of countries had no data for marriage by age 15 and 18 respectively; and 16 and 15 of the countries have no significant trends (p-value > 0.10).

### Sexual violence experienced by women before the ages before age 15, 18 and 22 years

The prevalence of sexual violence before ages 15, 18, or 22 years varies widely across countries, ranging from 0.1 to 21.7. These indicators had no data in 89 countries (65%), or have less than two data points in 39 (28%), and only 9 countries have enough data to possibly assess the trends.

#### Highest prevalence

These indicators began to be collected in 2006. Fifteen years ago, the highest rates were in Mali (y_2006_ = 10), Uganda (y_2006_ = 8), and Gabon (y_2006_ = 6) for before age 15; for age 18, the highest rates were in Uganda (y_2006_ = 17), Cameroon (y_2011_ = 15), and Congo Dem. Rep. (y_2014_ = 12); and for age 22, the highest rates were in Uganda (y_2006_ = 22), Cameroon (y_2011_ = 19), and Congo Dem. Rep. (y_2014_ = 18). Many countries with available data have seen a reduction in these rates from 2018–2020, but not all countries have the current data (Table 5). None of the countries below have more than one data point to observe the progress over time.

**Table 5:** Highest prevalence rates for sexual violence experienced by women before ages 15, 18, or 22^a^

Country	Income Category	Prevalence rate in	Year	Category based on the Prevalence
**- Sexual violence before 15**				
Gabon	Upper-middle	6,4	2012	high
Congo, Dem. Rep	Low	3,9	2014	medium
Honduras	Low-middle	3,6	2014	medium
Rwanda	Low	3,6	2020	medium
Gambia, The	Low	3,0	2020	medium
Liberia	Low	2,6	2020	medium
**- Sexual violence before 18**				
Congo, Dem. Rep	Low	11,7	2014	high
Rwanda	Low	10,2	2020	high
Gabon	Upper-middle	9,7	2012	high
Ghana	Low-middle	8,3	2008	high
Liberia	Low	5,0	2020	medium
Gambia, The	Low	4,8	2020	medium
**- Sexual violence before 22**				
Congo, Dem. Rep	Low	18,2	2014	high
Rwanda	Low	16,0	2020	high
Ghana	Low-middle	13,5	2008	high
Gabon	Upper-middle	11,2	2012	medium
Gambia, The	Low	6,6	2020	medium
Liberia	Low	6,4	2020	medium

^a^the highest rates are presented in six countries: three countries with the available year (in red) and three with recent rates for 2020

#### Trends

**Fig. 1:**
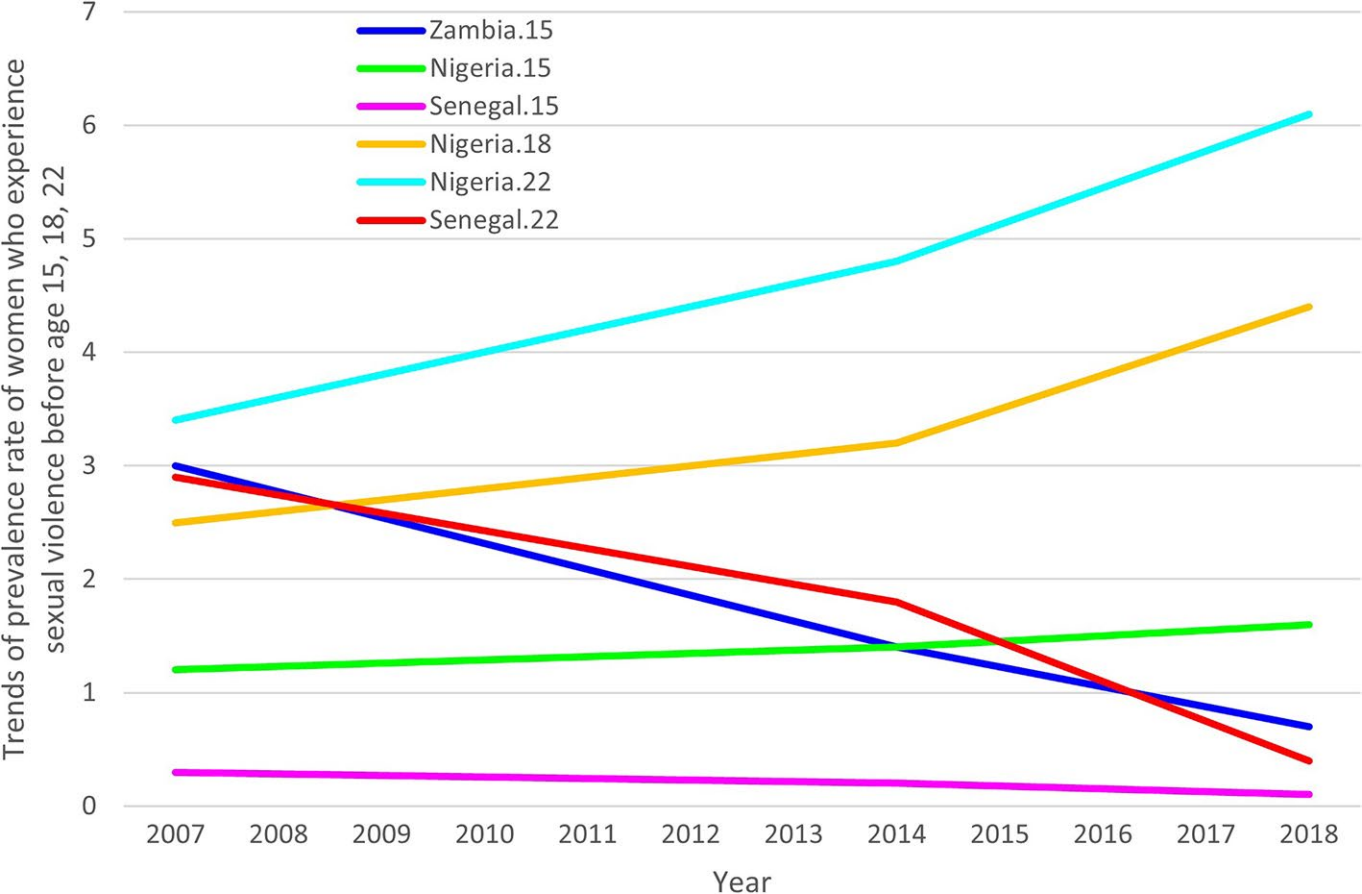
Trends for women who experienced first sexual violence before age 15, 18, and 22 (of women ages 15–49)

Trend analysis was conducted for nine countries with at least three data points: Haiti, Mali, Nigeria, Philippines, Rwanda, Senegal, Uganda, Zambia, and Zimbabwe. Among these, only Nigeria and Senegal showed a significant trend. Zambia and Senegal had a decline while Nigeria displayed increasing trends,. The remaining countries had a p-value > 0.10 (Fig. 1).

When observing prevalence rate data without making statements of trends (less than 2 data points), it is evident that most countries have reduced the rates in every indicator, including Cameroon, Dominican Republic, Haiti, Kenya, Malawi, Mozambique, Senegal, Uganda, and Zambia, for every indicator. Some countries have increased rates in three indicators, such as Gambia, Liberia, Nigeria, Tanzania, and Timor-Leste. India, Nepal, Sierra Leone, and Rwanda have increased rates for before 18 and 22, but for the before 15 indicator, Sierra Leone decreased, while India, Nepal, and Rwanda remained the same.

### Early sexual intercourse before age 15 among women and men

Many countries have shown significant progress towards reducing child marriage rates, with some exceptions. In Latin America, some countries have demonstrated an increasing trend (Table 6). Among the data of women, out of 137 LMIC countries, 100 countries had absence of data. Among the 37 countries with available data, 19 countries have a non-significant p-value to show a trend, while 18 show a trend: 6 with an increasing trend and 12 with a decreasing one. For men, 107 countries do not have any data. Among the 30 with available data, only 10 have significant data indicating a trend. Out of these, 9 show a decreasing trend, and only 1 has an increasing trend (Additional file 1:Appendix 3 and Appendix 4).

**Table 6:** Highest prevalence rates for women and men who initiated sexual intercourse before 15 (of ages 15–19)

Country	Income Category	Prevalence rate in	Year	Category based on the Prevalence
**- Women**				
Mozambique	Low	24,5	2015	high
Congo, Rep	Low	23,0	2012	high
Angola	Low-middle	22,9	2016	high
Niger	Low-middle	22,8	2012	high
Cote d'Ivoire	Low	20,8	2012	high
**- Men**				
Angola	Low-middle	34,8	2016	high
Gabon	Upper-middle	34,6	2012	high
Haiti	Low-middle	31,6	2017	high
Colombia	Upper-middle	30,5	2015	high
Dominican Republic	Upper-middle	28,3	2013	high

#### Highest prevalence

Thirty years ago, the highest rates of of sexual intercourse before age 15 for women were in Cote d'Ivoire (y_1994_ = 32), Niger (y_1992_ = 30), and Mozambique (y_1997_ = 29), while the highest rates for men were in Zambia (y_1996_ = 39), Brazil (y_1992_ = 34), and Kenya (y_1998_ = 32). These rates have now reduced now by an average of 23 for women and 11 for men. Currently, the countries with highest prevalence for women are Mozambique, Rep. Congo, Angola, Niger and Cote d'Ivoire, while for men are Angola, Gabon, Haiti, Colombia, and Dominican Republic.

#### Trends

Among the countries with highest increasing trends, Colombia, Dominican Republic and Bolivia have tansitioned from low to a medium value for women, and Liberia showed a small change for men. There was a significant reduction within the top 10 countries with higher prevalence declining from 26 to 19% for women and from 27 to 21% for men, with both cases transitioning from high prevalence to a medium level. There are also increasing trends with an average CAGR of 3.22 in 6 countries for women and 0.69 for men in 1 country (Table 7).

**Table 7:** Highest rates of women and men who initiated sexual intercourse before 15 (of ages 15–19), p-value ≤ 0.10

Country	Income Category	Coefficient	p-val	Sig	Start Rate (yo)	End Rate (ye)	Start Year	End Year	Category based on end value
**- Women**									
**Increasing trend**									
Colombia	Upper-middle	**0.4**	0.001	*	5.6	15.5	1990	2015	medium
Dominican Republic	Upper-middle	**0.35**	0.006	*	7.8	16.8	1991	2013	high
Bolivia	Low-middle	**0.21**	0.046	*	4.6	7.3	1994	2008	low
**Decreasing trend**									
Uganda	Low	**-0.57**	0.057	*	23.8	10.3	1995	2016	medium
Cameroon	Low-middle	**-0.51**	0.026	*	23.6	11.9	1991	2018	medium
Sierra Leone	Low	**-0.5**	0.067	*	22.3	16.8	2008	2019	high
**No trend**									
Mozambique	Low	-0.32	0.172		28.6	24.5	1997	2015	high
Niger	Low	0.14	0.907		29.8	22.8	1992	2012	high
Cote d'Ivoire	Low-middle	-0.55	0.213		31.9	20.8	1994	2012	high
**- Men**									
**Increasing trend**									
Liberia	Low	**0.06**	0.063	*	8.6	9.4	2007	2020	low
**Decreasing trend**									
Namibia	Upper-middle	**-1.39**	0.099	*	31.3	13.4	2000	2013	medium
Zambia	Low	**-0.98**	0.054	*	39.3	16.3	1996	2018	medium
Kenya	Low-middle	**-0.84**	0.037	*	31.7	19.6	1998	2014	medium
**No trend**									
Haiti	Low-middle	0.52	0.455		20.1	31.6	1995	2017	high
Dominican Republic	Upper-middle	0.44	0.241		23.0	28.3	1996	2013	high
Mozambique	Low	-0.16	0.782		23.5	26.5	1997	2015	high

#### No trend

Some countries have shown no progress over time, and in these cases, some have even higher prevalence rates at (y_e_), such as Mozambique, Niger and Cote d’Ivoire, and Liberia for women, and Haiti, Dominican Republic, Mozambique or Lesotho for men.

## Discussion

The present study investigates trends in the prevalence of child marriage, sexual violence and early sexual intercourse among LMIC over the past three decades. The results indicate a decline of these indicators in most countries, regardless of their income group. However, a small number of countries show small increasing trends in child marriage and first sexual intercourse. The findings reveal that in the past 30 years, the highest prevalence for marriage by 15 years has reduced from 23% in ≈1994 to 19% ≈2016, and the marriage by age 18 reduced from 59% in to 43% among the top 20. Additionally, the prevalence of sexual violence showed declining trends for Zambia and Senegal while Nigeria had an increasing trend. And for early sexual intercourse we can observe declining trends going from 26 to 19% for women and from 27 to 21% for men, shifting the prevalence from high to medium. Conversely, some countries experienced an increasing trend, like Colombia, Dominican Republic, and Bolivia for women, while for men, Liberia showed an increasing trend. Additionally, there are countries with no statistical trends or showing no significant relation of change over time or even have no data to assess their progress, particularly for men.

Although we did not assess a causal relationship between these three indicators, we can observe a pattern. For instance, countries like Nigeria, Senegal and Zambia experienced significant reductions in child marriage, early sexual intercourse, and sexual violence rates. However, the sexual violence is rising in Nigeria because of an increase on social conflicts and Internally Displaced Persons (IDPs) [14, 47]. Simultaneously, it was evidenced that not all countries have reduced the prevalence rates equally. While some countries experienced a high progress others experienced a low one. This may be due to different risk factors, some persistent like education, unequal income distribution, law enforcement, and other recent like armed conflicts or natural disasters that lead to economic shocks. First factor is education and other related factors. Limited education, deep-rooted cultural norms, lack of awareness or the reinforcement of the law, particularly in rural areas worsen the problem, necessitating immediate and focused interventions for meaningful change. A recent study examining progress in child marriage between 1990 and 2017 reported similar findings, revealing a higher prevalence of child marriage and slower progress in reducing it in sub-Saharan Africa and South Asia [29]; other studies indicated that child marriage is related to the employment opportunities, exposure to health information through mass media, and gender and social equality [46]. Second, factor is the unequal income distribution. A study conducted in Bangladesh, India, Indonesia, and Nepal revealed that education, wealth, and residence-based inequalities play a significant role in child marriage, with higher incidence is observed among disadvantaged groups [30] or the poor [16, 24, 38]. Niger and Chad are ranked among the porest countries in the world which pushes families to turn to child marriage as a protective measure against the crisis [29]. A third factor is the armed conflicts or natural disasters that create economic shocks. The Central African Republic has an armed conflict that could be related to forced sexual debut, sexual violence [9]; the case of Indonesia with a natural disaster; the influence of COVID-19 pandemic, which generated poverty and social strain [35] and may have increased child marriage. Therefore, it is important to further investigate the reasons why there are some increasing trends observing which are the persistent social habits based inequality and observing those vulnerable populations within or across countries.

Interventions could base in those effective actions and protective factors. Education has proven to be the means to reduce the incidence [38]; but there are still many countries that have not effectively implemented comprehensive sexuality education or engaged in multisectoral collaboration to address the problem [12]. A range of interventions indicated above can be implemented [4, 7]. Nonetheless, it is important to think that the simple creation and implementation does not necessarily solve the problem. For instance, the creation or improvement of laws to prevent child marriage is an effective action like reported in Ethiopia [25, 34], but needs to have enforcement, adequate implementation, or marriage registers to evidence efforts [48]. Successful examples of programs that employ a multisectoral approach and focus on behavioural change are the Ishraq program in Egypt, the Berhane Hewan program in Ethiopia, the Adolescent Participatory Project in Nepal, and in India, the Development Initiative Supporting Healthy Adolescents (DISHA), the Maharashtra Life Skills Program [37], and the Promoting Change in Reproductive Behavior (PRACHAR) program [41]. These success examples can be used to drive public health policy and programs worldwide [28, 30, 39], as it is suggested and done by the UNFPA-UNICEF Global Program to End Child Marriage which continuously shows progress (UNFPA-UNICEF, 2023). An economic strategy to reduce the financial burden of caring for a family, especially in vulnerable areas with limited opportunities for education, labor market access, or other societal roles [6, 21, 40].

An important aspect highlighted by this research is the lack of data, particularly in countries without monitoring programs, making it difficult to assess the situation and implement suitable policies to reduce child marriage and other harm practices. The scarcity of sex and age disaggregated data perpetuates inequality, as at-risk populations cannot be identified [10]. This hamper understanding behavioural patterns, interventions efficacy, causes, effects, and priorities. The absence of robust monitoring and reporting systems can limit effective policy-making and tailored implementations. Sharing and learning from experiences across countries can be beneficial [32]. There is a call for standardizing gender and age-disaggregated data related to child marriage, which can allow us to focus on adolescents with accurate analysis, identification of high-risk areas, and evaluation of the effectiveness of interventions [10]. Consistent data empowers organizations and governments to implement tailored strategies, allocating resources efficiently.

This study has notable strengths. It analyzes the prevalence for various indicators across different data points allows examination of temporal trends in child marriage dynamics. Focusing on vulnerable age groups, especially girls under 15 and 18, provides targeted insights into their unique challenges. Multiple indicators related to child marriage contribute to a comprehensive understanding using two methods, trend analysis and CAGR. We opted for CAGR as another way to analyse the decline of the prevalence per year in addition to coefficients of the regression analysis. Rigorous statistical analysis strengthens the credibility and scientific rigor of the findings. However, the study also has limitations, such as the lack of disaggregated age and sex data, and continuous data for all countries. Another limitation is the dataset used may have errors in the way indicators are framed or projected. The authors tried to mitigate this by observing trends with a p-value ≤ 0.10 and analyzing prevalence rates cross-sectionally or comparatively with other published papers to further understand the problem but not all countries were represented in publications. Future studies could focus on the increasing or declining trends in short or longer periods to identify strategies that can be replicated and scaled up as well as the barriers to progress. Another limitation is the absence of micro-level data; future studies could explore relations with other indicators like income inequality, teenage pregnancy or assess the costs of child marriage in different contexts to develop policy strategies [12, 15], or focus on the relation with violence against children.

In conclusion, the results show that there is a substantial decline in child marriage, and early sexual intercourse in most countries independent from the income group. Only a few countries show slight increasing trends. The improvements confirm that policies that address education, employment, and deep-rooted gender inequality at societal level seem to be effective and help reach the SDG. However, better data are needed to enhance the understanding of the development of child marriage in these and other countries to improve the effectiveness of policy intervention. Therefore, we recommend that policymakers not only include existing evidence that continues progress, but also increase and improve the monitoring of relevant indicators.

### Supplementary Information

**Additional file 1:** **Appendix 1.** Women who were first married by age 15 (% of women ages 20-24). **Appendix 2.** Women who were first married by age 18 (% of women ages 20-24). **Appendix 3.** Women who initiated sexual intercourse before age 15 (% of women ages 15-19. **Appendix 4.** Men who initiated sexual intercourse before age 15 (% of women ages 15-19). **Appendix 5.**

## Data Availability

The datasets used and/or analysed during the current study are available from the corresponding author on reasonable request.

## Abbreviations

LMICs: Low-and middle-income countries
SDG: Sustainable Development Goals
